# The effect of midline shift on survival time in dogs with structural brain disease diagnosed on MRI

**DOI:** 10.1111/vru.13450

**Published:** 2024-10-10

**Authors:** Bethany Guy, Paul Freeman, Sam Khan, Marie‐Aude Genain

**Affiliations:** ^1^ Department of Veterinary Medicine Queen's Veterinary School Small Animal Hospital, University of Cambridge Cambridge UK

**Keywords:** diagnostic imaging, intracranial pressure, neurology, seizures

## Abstract

The effect of midline shift identified on brain MRI on survival time in dogs with structural brain disease is relatively unknown. This retrospective single‐centered cohort study reviewed medical and imaging data of 77 dogs with structural brain lesions evident on MRI. Images were reviewed for the presence of midline shift, brain edema, foramen magnum herniation, and ventriculomegaly. Kaplan–Meier method and Cox regression analysis were undertaken to compare survival between dogs with and without midline shift. Midline shift was present in 40 of 77 (52%) dogs and absent in 37 of 77 (48%). Univariate analysis revealed that dogs with midline shift had a median survival time of 34.5 days (95% CI, 4–108 days) compared with 241 days (95% CI, 133,‐ days) in dogs without midline shift (hazard ratio = 2.67, 95% CI, 1.5–4.49). Multivariate Cox regression analysis revealed a hazard ratio of 3.6 (95% CI, 1.7–7.6; *P*‐value < .001) for dogs with midline shift. Shorter median survival times remained significant in all groups after segregation based on etiological diagnosis. The significantly shorter survival times observed herein for dogs with midline shifts, regardless of etiologic cause, provide further evidence that midline shift holds value as a negative prognostic factor in diagnostic imaging.

## 1 INTRODUCTION

The displacement of anatomical structures due to space‐occupying pathology throughout the body is termed “mass effect”.^1^, ^2^ When mass effect occurs specifically within the cranium and results in the displacement of structures laterally across the midline, altering the normally symmetrical structure of the canine brain, this is known as midline shift.^1^ Mass effect resulting in midline shift can be followed by herniation, brainstem compression, and death.^1^ Most of the current knowledge regarding midline shift and prognosis or survival times has been elicited from the human literature.^3^, ^4^, ^5^, ^6^, ^7^, ^8^, ^9^, ^10^, ^11^, ^12^, ^13^, ^14^, ^15^, ^16^, ^17^, ^18^, ^19^, ^20^, ^21^, ^22^, ^23^, ^24^, ^25^ Midline shift due to mass effect occurs when there is asymmetrical pathology causing lateral deviation of intracranial structures across the midline and is significantly associated with death.^1^, ^3^, ^4^ Human studies highlight the influence of midline shift on survival across various neurological conditions, including malignant ischemia, intracranial infarction, malignant gliomas, and intracranial hematomas; it is also an important factor to be considered in the neurological status and predicting prognosis in patients with extradural hematomas and the probability of developing late posttraumatic seizures.^5^, ^6^, ^7^, ^8^, ^9^, ^10^, ^11^, ^12^, ^13^, ^14^, ^15^, ^16^, ^17^, ^18^, ^19^, ^20^, ^21^ Identifying midline shifts and the degree of shift may influence the treatment protocol chosen by a clinician.^20^, ^21^, ^22^, ^23^ Despite midline shift decreasing after decompressive surgery in patients with ischemic stroke, this does not always lead to fewer deaths; a midline shift of less than 5 mm postsurgery correlates significantly with favorable long‐term functional outcomes.^24^, ^25^ Prognostic factors associated with poor outcomes in humans include neurological diagnosis, a lower Glasgow Coma Score, increasing age, midline shift, brain herniation, lesion volume, lesion location, cerebral contusion, hypertension or hypotension, hypopnoea or hyperpnoea, hyperglycemia, and hypoxemia.^5^, ^6^, ^7^, ^8^, ^9^, ^10^, ^11^, ^12^, ^13^, ^14^, ^15^, ^16^, ^17^, ^18^, ^19^, ^20^, ^21^, ^22^, ^23^, ^24^, ^25^ It is also important, however, to consider that survival in neurological patients does not always correlate with normal neurological status or a good quality of life.

In veterinary literature, there is a shortage of information regarding the effects of midline shift in dogs. The current knowledge identifies differences in whether midline shift affects survival time when considering diagnosis and treatment protocols.^26^, ^27^, ^28^, ^29^, ^30^ Midline shift was not associated with shorter survival times in 52 dogs with meningoencephalitis.^26^ However, there was a substantial difference in initial short‐term survival, and a trend suggested dogs with a larger midline shift on MRI have a poorer prognosis.^26^ A negative correlation was identified between midline shift and survival time in dogs with meningioma following surgical resection alone, unlike for glioma, where no such association was found.^27^ A negative correlation between midline shift and outcome score has been identified using MRI in dogs after traumatic brain disease.^28^ In contrast, early CT in dogs with traumatic brain injury midline shift was not associated with survival to discharge.^29^ Midline shift was apparent in 52% (14/27) of dogs with head trauma^30^; it was not associated with survival times, therefore, differing from findings in foxes and most human studies.^4^, ^6^, ^10^, ^31^, ^32^

Advanced imaging is valuable in identifying midline shifts in dogs.^26^, ^27^, ^28^, ^29^, ^30^ MRI can aid in distinguishing neoplasia and inflammatory brain disease in dogs; however, due to similar imaging features, misclassification of brain disease can occur, and therefore, it is unknown whether the etiology contributing to midline shift affects survival time.^33^, ^34^

From the current literature, the effect of midline shift on the survival time and neurological status in dogs with structural brain disease is unclear. This information is important as it may aid owners in making informed decisions and influence the treatment protocol chosen by a clinician. We hypothesized that dogs with a structural brain disease conspicuous on MRI evaluation with a midline shift would have a shorter survival time compared with dogs with no midline shift.

## 2 MATERIALS AND METHODS

### 2.1 Selection and description of subjects

Medical records were retrospectively reviewed for dogs presented to the Queen's Veterinary School Hospital (QVSH) at the University of Cambridge between January 2017 and December 2021 who had undergone brain MRI. Cases were identified utilizing the imaging database and reviewed to ensure they met the inclusion criteria by a first‐year diagnostic imaging resident (B.G.). Study approval was granted by the University of Cambridge Department of Veterinary Medicine Ethics and Welfare Committee (Approval CR613). Inclusion criteria were a structural brain lesion evident on MRI which included at least T1W spin echo (SE) or T2W fast spin echo (FSE) sagittal, transverse, and dorsal sequences. A structural brain lesion was included if it fell within the recognized causes of structural epilepsy as defined by the international veterinary epilepsy task force.^35^ Dogs were excluded if a conclusive diagnosis was not obtained (based on MRI and laboratory results) because of incomplete medical or imaging data, if no structural brain disease was evident, or if an extracranial disease with an intracranial component was identified. Dogs were subsequently divided into the following categories based upon diagnosis: neoplasia, inflammatory, and other, according to documented classifications found in the literature.^33^, ^34^

### 2.2 Data recording and analysis

Data collected included the age, sex, breed, mentation on presentation, history of seizures, diagnosis, and survival time. Mentation on presentation was categorized as normal or abnormal based on neurological examinations performed by clinicians with a range of experience, from interns to ECVN diplomates. The diagnosis was classified as definitive if a postmortem evaluation and histopathology results were available or presumptive if based on history, clinical signs, physical and neurological examinations, MRI evaluation, and CSF analysis. If dogs were euthanized attributable to a diagnosis that was unrelated to their neurological diagnosis, this was recorded. Survival times were obtained by reviewing medical records and contacting referring practices and owners either by email or telephone. If a dog was euthanized or died on the day of diagnosis, this was recorded as day 0; if they were euthanized the following day, then this was recorded as a survival time of 1 day. Dogs still alive on the 11th of August 2022 were censored.

MR images were anonymized and blindly reviewed retrospectively by an ECVDI‐certified veterinary radiologist (M‐A.G.; Horos version 3.3.6, Purview; iMac Retina 4K, Apple Inc.). Imaging was performed using three MR systems, which varied in field strength. These include two utilizing low‐field, permanent magnets of 0.25 T (Esaote VetMR Grande, 69 studies) and 0.18 T (Esaote VetMR, Genova, Italy, 1 study) and one utilizing a high‐field, superconducting 1.5 T magnet (Philips Achieva, Philips Healthcare, 7 studies). Coil choice and technical factors varied according to standard protocols and available equipment at each study location. Dogs were positioned in sternal recumbency in the low‐field magnets and dorsal recumbency in the high‐field magnet. Echo time, repetition time, slice gap, and interslice gap are included in Appendix 1. A standard brain protocol at the QVSH was performed in most of the dogs which included 2D T2W FSE transverse and sagittal sequences, T1W SE, FLAIR, and gradient echo (GE) transverse sequences, which include T2W GE sequences for the images acquired using the low field permanent magnets and T2* sequences when using the high field superconducting magnet, T1W SE dorsal sequence and postcontrast T1W SE dorsal and transverse sequences. In two included patients, complete examinations were not available for review, with omission of T2W sagittal images in one case, and lack of T2W imaging in any plane in the other. Images were assessed for the presence or absence of midline shift, brain oedema, foramen magnum herniation, and ventriculomegaly.

### 2.3 Statistics

Analyses of the data were performed by one author (S.K.) and the veterinary diagnostic imaging resident (B.G.), using spreadsheet (Microsoft Excel, Microsoft Corporation, Thames Valley Park) and statistical analysis software (R Studio Team [2020], RStudio: Integrated Development for R. RStudio, PBC, URL: http://www.rstudio.com/). The Kaplan–Meier method and Cox regression analysis were undertaken to compare survival between dogs with and without midline shift. Dogs were subsequently segregated by diagnosis into two groups, neoplasia and nonneoplastic groups, which included the inflammatory and “other” categories, and the analysis was repeated. A multivariate Cox regression analysis was undertaken to also include brain edema, foramen magnum herniation, or ventriculomegaly. A *P*‐value < .05 was considered statistically significant.

## 3 RESULTS

**FIGURE 1:**
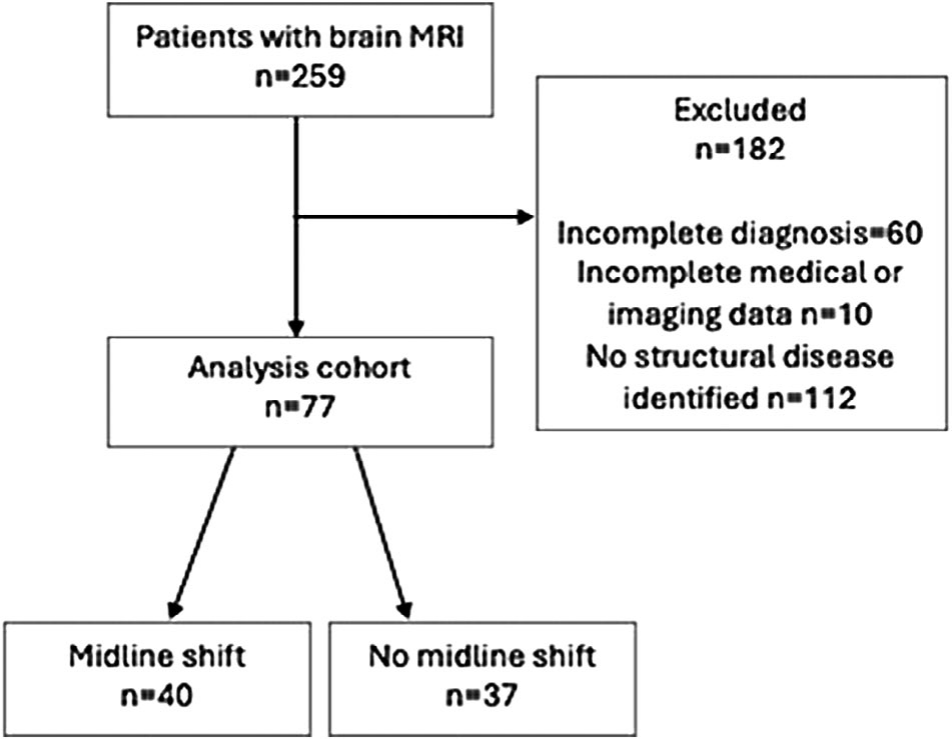
The flow diagram illustrates the number of dogs included and excluded in the study.

A total of 259 dogs had a brain MRI between January 2017 and December 2021. Dogs were excluded because of an incomplete diagnosis (60), incomplete medical or imaging data (10), and because of a diagnosis unrelated to intracranial structural brain disease (112; Figure 1). Excluded dogs with incomplete medical or imaging data comprised of dogs with an unknown survival time (5), incomplete imaging data (4), or incomplete medical data (1).

A total of 77 dogs were included in the study. Crossbreed (*n* = 11), French Bulldog (*n* = 10), Staffordshire Bull Terrier (*n* = 7), and Jack Russell Terrier (*n* = 6) were the most common breeds. Sexes included: 34 female (25 neutered) and 43 male (32 neutered). The median age was 8 years (IQR, 4–10 years).

Dogs were categorized based on their imaging diagnosis: neoplasia (48), inflammatory (15), and other (14). In the neoplasia category, 26 dogs had intra‐axial tumors, and 22 dogs had extra‐axial tumors, which included pituitary tumors (10) and intraventricular tumors (3). Forty‐four dogs with neoplasia had single tumors and four had multifocal diseases. Of the dogs with multifocal disease, two were extra‐axial and two intra‐axial in location. The other category included cases of hydrocephalus and supracollicular fluid accumulation or both (5), ischaemic infarction (3), Chiari‐Like malformation (3), porencephaly (2), and brain trauma (1). A difference was identified between the ages, sex, neutered status, and most common breeds represented in the neoplasia group and nonneoplastic group Table 1.

**TABLE 1:** Demonstrates the median and interquartile age ranges, sex including neutered status, and most common breeds represented for the two groups.

	Neoplasia group	Nonneoplastic group
Age (years)	Median = 9.7 (IQR 8.0–10.0)	Median = 3.7 (IQR 2.8–4.8)
Sex	Female *n* = 21 (18 neutered) Male *n* = 27 (22 neutered)	Female *n* = 13 (7 neutered) Male *n* = 16 (10 neutered)
Most represented breeds in each category, including the number of dogs when compared with the total number of dogs in each breed	Crossbreed (8/11), Staffordshire Bull Terrier (7/7) French Bulldog (6/10) Jack Russell Terrier (3/6)	Inflammatory: Chihuahua (3/5) Pug (2/4) Jack Russell Terrier (3/6) Other: No predilection

Sixteen dogs were still alive at the date of censoring (August 11, 2022). Of these dogs, 13 (81.3%) had no evidence of midline shift on MRI. Diagnoses included neoplasia; intra‐axial (2), pituitary macroadenoma (3); inflammatory (2); and eleven other diagnoses including Chiari‐Like malformation (3), hydrocephalus (2), ischaemic infarction (2), supracollicular fluid accumulation and hydrocephalus (1), and porencephaly (1). At the time of censoring, the minimum follow‐up time was 233 days (intra‐axial neoplasia), and the maximum follow‐up time was 2048 days (supracollicular fluid accumulation and hydrocephalus).

A definitive diagnosis was acquired in 10 dogs, and all were consistent with their imaging diagnosis category. One dog was euthanized because of suspected idiopathic pulmonary fibrosis 90 days following an initial diagnosis of a pituitary macroadenoma.

**FIGURE 2:**
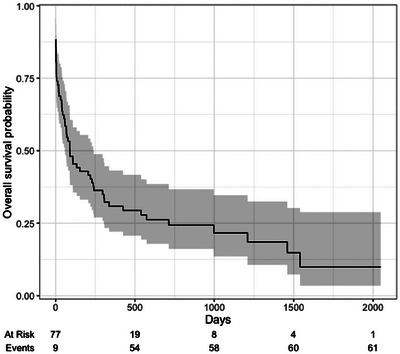
Kaplan–Meier curve demonstrating the overall survival probability for dogs with structural brain disease (*n* = 77). The overall survival probability is a fraction of the total number of dogs still alive. The numbers below denote the number of dogs at risk of dying at each time point “at risk” and the number of deaths that have occurred “events”. The shaded area represents the 95% confidence interval.

**FIGURE 3:**
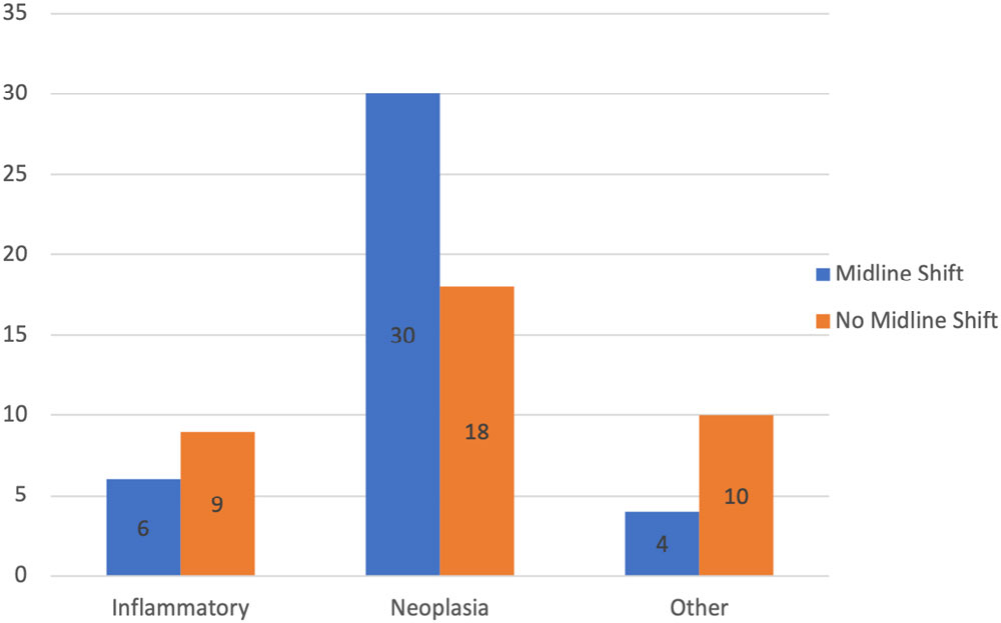
Bar chart demonstrating the number of dogs in each diagnosis category with and without midline shift.

**FIGURE 4:**
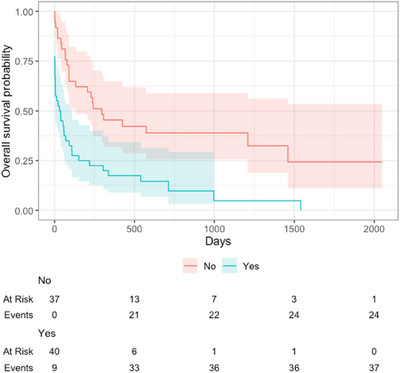
Kaplan–Meier curve demonstrating the overall survival probability for dogs with structural brain disease. No, indicates the number of dogs without midline shift, *n* = 37. Yes, indicates the number of dogs with midline shift *n* = 40. The overall survival probability is a fraction of the total number of dogs still alive within each of the two groups. The numbers below denote the number of dogs at risk of dying at each time point ‘at risk’ and the number of deaths that have occurred “events”. The shaded area represents the 95% confidence interval.

The median survival time for the total population was 90 days (95% CI, 58–241 days; Figure 2). Midline shift was present in 40 of 77 (52%) dogs, including 30 dogs diagnosed with neoplasia, 6 with inflammatory disease, and 4 with other diseases (Figure 3). Other diseases included two dogs with a diagnosis of porencephaly, which was causing mass effect with midline shift away from the lesion rather than midline shift toward the lesion due to loss of brain parenchyma, as would normally be expected with porencephaly, one traumatic brain injury secondary to a dog fight and one dog with an ischemic vascular event. The median survival time in dogs with midline shifts was 34.5 days (95% CI, 4–108 days) in comparison to 241 days (95% CI, 133,‐ days) in dogs without midline shifts (Figure 4). Univariate analysis showed that dogs with structural brain disease identified on MRI were 2.67 times more likely to die if they had midline shift (Hazard ratio = 2.67, 95% CI, 1.5–4.49, *z* value = 3.68, *P*‐value = < .01; Figure 4).

**FIGURE 5:**
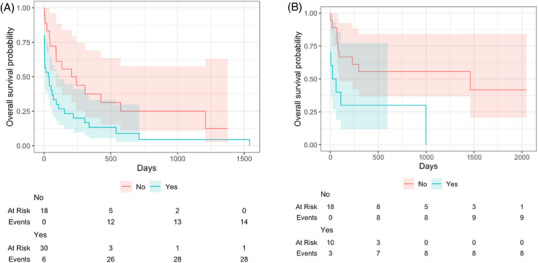
(A) Kaplan–Meier curve demonstrating the overall survival probability for the neoplasia group. No, indicates the number of dogs without midline shift, *n* = 18. Yes, indicates the number of dogs with midline shift *n* = 30. (B) Kaplan–Meier curve demonstrating the overall survival probability for the nonneoplastic group. No, indicates the number of dogs without midline shift, *n* = 19. Yes, indicates the number of dogs with midline shift *n* = 10. The overall survival probability is a fraction of the total number of dogs still alive within each of the two groups. The numbers below denote the number of dogs at risk of dying at each time point “at risk” and the number of deaths that have occurred “events”. The shaded area represents the 95% confidence interval.

Median survival time in the neoplasia group with midline shift was 35 days (95% CI, 4–109 days) and 224 days without midline shift (95% CI, 89,‐days). Dogs diagnosed with neoplasia were 2.15 times more likely to die if they had midline shifts (HR = 2.15, 95% CI, 1.12–4.11, *P*‐value = .021; Figure 5A). Median survival time in the nonneoplastic group with midline shift was 40 days (0, ‐days) and 1460 days without midline shift (95% CI 89,‐). Dogs with inflammatory or other diagnoses were 3.01 times more likely to die if they had midline shifts (HR = 3.01; 95% CI, 1.12–8.10; *P*‐value = .029; Figure 5B).

Multivariate Cox regression analysis revealed a hazard ratio of 3.6 (95% CI, 1.7–7.6; *P*‐value < .001) for dogs with midline shift. Hazard ratios for other variables analyzed are presented in Table 2.

**TABLE 2:** Results for the multivariate Cox regression analysis accounting for the effect of imaging covariates on the midline shift and no midline shift survival curves.

Characteristic	Hazard ratio	95% CI	*P*‐value
Midline shift^*^	3.58	1.70, 7.55	<.001
Brain oedema	0.67	0.31, 1.45	.3
Foramen magnum herniation	1.47	0.45, 4.82	.5
Ventriculomegaly	1.12	0.67, 1.86	.7

^*^The significant result. Significance is defined as a *P*‐value of < .05.

More dogs with midline shifts presented with seizures than dogs without midline shifts, 68% (27/40) dogs as compared with 38% (14/37) dogs, respectively. Abnormal mentation was similar in the two categories: 55% (22/40) of dogs with midline shift and 62% (23/37) of dogs without midline shift, respectively.

Eight dogs with midline shift were euthanized on the day of diagnosis and one the following day. One dog died on the day of diagnosis. These dogs were diagnosed with neoplasia in seven cases and one case of each of the following: meningoencephalitis of unknown origin, brain trauma following a dog fight, and porencephaly. Six of the dogs had a history of seizures, and five had abnormal mentation on arrival at the QVSH. One patient without a midline shift, diagnosed with neoplasia, was euthanized the following day. This dog presented with abnormal mentation and a history of seizures.

## 4 DISCUSSION

The results of this study suggest that dogs with a midline shift secondary to intracranial structural brain disease, conspicuous on MRI evaluation, have a shorter survival time compared with dogs without midline shift (35 vs 241 days). The hazard ratios for both the univariate analysis (hazard ratio = 2.665, 95% CI, 1.5–4.49) and multivariate analysis (hazard ratio 3.6, 95% CI, 1.7–7.6; *P*‐value < .001) emphasize this. When separated by diagnosis, we found similar differences in survival times for those with and without midline shift. Multivariate analysis to identify factors contributing to increased risk of death reiterated the significance of midline shift, whereas brain edema, foramen magnum herniation, and ventriculomegaly did not achieve significance.

The results from this study agree with Suñol et al.^27^ where a negative correlation was identified between midline shift and survival time in dogs who underwent surgical resection of meningiomas. They are also in agreement with Beltran et al.^28^ in which a negative outcome score is correlated with midline shift in dogs with brain trauma. However, they are dissimilar to Wyatt et al.^29^ and Chai et al.^30^ in which dogs with midline shift following traumatic brain injury were not associated with survival to discharge. Only one dog with brain trauma was included in our study, and therefore, the comparisons should be interpreted with caution. It is also important to highlight that more dogs were diagnosed with neoplasia (*n* = 48) than inflammatory or other structural brain diseases (*n* = 29), and it is, therefore, unclear whether these findings would apply to a larger cohort of dogs with other diagnoses.

In this study cohort, regardless of diagnosis, survival times were similar, suggesting that midline shift is relevant for all categories of diagnosis. It is important to acknowledge, however, that 87% (67/77) of the dogs had a presumptive diagnosis. MRI sensitivity and specificity for both detection of brain lesions (Se: 94.4%, Sp: 95.5%) and classification of these lesions by etiology in the absence of clinical data has been recognized to be high, neoplasia (Se: 87.4%, Sp: 91.7%) and inflammatory brain disease (Se: 86.0%, Sp: 93.1%), though with reduced sensitivity in the case of cerebrovascular disease (Se: 38.9%, Sp 97.7%).^33^ In dogs with a leading diagnosis of glioma 10/148 (6.8%) were histologically identified as granulomas.^36^ Good correlation was, however, observed between the imaging diagnosis and definitive diagnosis obtained in 10 dogs in the study: all dogs were correctly assigned into the three categories.

Treatment protocols vary depending on diagnosis; despite the difference, in this population of dogs, survival times were similar for all categories of diagnosis.

Many of the limitations of this study are due to being retrospective, which has led to many dogs being excluded because of incomplete records, a greater number of dogs with a diagnosis of neoplasia, nonstandardized imaging, treatment protocols, and unstandardized follow‐up, each of which is a potential source of bias. A greater proportion of dogs with neoplasia and midline shift were also euthanized within 24 h of diagnosis compared with those without, and it is unclear if this was based upon clinical deterioration or a perception of a poorer prognosis and, therefore, could bias survival times.

In conclusion, dogs in this cohort with midline shift secondary to structural brain disease on brain MRI had a shorter survival time than dogs without midline shift regardless of diagnosis. Due to the small number of dogs with varying diagnoses in the other category, the conclusion is most relevant for dogs diagnosed with neoplastic and inflammatory disease, and therefore, this should be considered when providing advice to owners. The results from this study provide additional information for clinicians and owners, which may influence advice and treatment options provided by clinicians. Further research, including a prospective study, focusing on the response to treatment and survival time in dogs with midline shift should be considered to validate these results.

## LIST OF AUTHOR CONTRIBUTIONS

### Category 1

- Conception and design: Guy, Freeman, Khan, Genain
- Acquisition of data: Guy, Genain
- Analysis and interpretation of data: Khan, Guy

### Category 2

- Drafting the article: Guy
- Revising article for intellectual content: Freeman, Khan, Genain, Guy

### Category 3

- Final approval of the completed article: Freeman, Khan, Genain, Guy

## CONFLICT OF INTEREST STATEMENT

The authors declare no conflict of interest.

## REPORTING CHECKLIST DISCLOSURE

STROBE checklist was used to direct manuscript construction.

## PREVIOUS PRESENTATIONS OR PUBLICATION DISCLOSURE

This manuscript has not been published or presented elsewhere and is not under consideration by any other journal.

## Supporting information



Supporting Information

## Data Availability

All data are available from the corresponding author upon reasonable request.
