# Supplementary material for: The effect of midline shift on survival time in dogs with structural brain disease diagnosed on MRI
Source: Vet Radiol Ultrasound. 2024 Oct 10;66(1):e13450. doi: 10.1111/vru.13450 (PMC11617613; doi:10.1111/vru.13450)
Supplement: Supplementary file 1 — Supporting Information [file VRU-66-0-s001.docx]

0.25T Esaote VetMR Grande

|  | TE (ms) | TR (ms) | Slice thickness (mm) | Interslice gap (mm) |
| --- | --- | --- | --- | --- |
| SE T1w sagittal | 26 | 350-1050 | 3-4 | 0.3-0.4 |
| FSE T2w sagittal | 90 | 2600-6630 | 3-4 | 0.3-0.4 |
| SE T1w transverse | 26 | 400-1050 | 3-4 | 0.3-0.4 |
| FSE T2w transverse | 90 | 2600-7780 | 3-4 | 0.3-0.4 |
| SE T1w dorsal | 26 | 250-960 | 3-4 | 0.3-0.4 |
| FSE T2w dorsal | 90 | 3750-6050 | 3-4 | 0.3-0.4 |

0.18T Esaote VetMR

|  | TE (ms) | TR (ms) | Slice thickness (mm) | Interslice gap (mm) |
| --- | --- | --- | --- | --- |
| SE T1w sagittal | 18 | 600 | 4 | 0.4 |
| SE T1w transverse | 18 | 600 | 5 | 0.5 |
| TSE T2w transverse | 80 | 3340 | 5 | 0.5 |
| SE T1w dorsal | 18 | 600 | 4 | 0.4 |
| TSE T2w dorsal | 80 | 2220 | 4 | 0.4 |

1.5T Philips Achieva

|  | TE (ms) | TR (ms) | Slice thickness (mm) | Interslice gap (mm) |
| --- | --- | --- | --- | --- |
| SE T1w sagittal | 10-15 | 400-641 | 3-3.5 | 0.3-0.35 |
| TSE T2w sagittal | 100 | 3027-4238 | 2-3.5 | 0.2-0.35 |
| TSE T1w transverse | 10-11 | 543-1066 | 2.5-4 | 0.25-0.4 |
| TSE T2w transverse | 90-100 | 3275-6320 | 2.5-4 | 0.25-0.4 |
| TSE T1w dorsal | 10-11 | 334-725 | 2.5-3.5 | 0.25-0.35 |
| TSE T2w dorsal | 90-100 | 2899-3427 | 3-4 | 0.3-0.4 |
